# Exploring the Impact of the COVID-19 Pandemic on Male Mental Health Emergencies Attended by Ambulances During the First National “Lockdown” in the East Midlands of the United Kingdom

**DOI:** 10.1177/15579883221082428

**Published:** 2022-03-04

**Authors:** Harriet Elizabeth Moore, Aloysius Niroshan Siriwardena, Mark Gussy, Bartholomew Hill, Frank Tanser, Robert Spaight

**Affiliations:** 1 DIRE Research Group, Department of Geography, University of Lincoln, Lincoln, UK^*^; 2 Community and Health Research Unit, School of Health and Social Care, University of Lincoln, Lincoln, UK^†^; 3 Lincoln Institute of Rural Health, University of Lincoln, Lincoln, UK; 4 Water WISER CDT, University of Loughborough, Loughborough, UK; 5 East Midlands Ambulance Service NHS Trust, Nottingham, UK; 6 EDGE Consortium^‡^

**Keywords:** male mental health, health inequality/disparity, health care issues, COVID-19, lockdown, ambulance mental health emergencies

## Abstract

The novel coronavirus disease 2019 (COVID-19) pandemic and associated mitigation strategies such as “lockdown” are having widespread adverse psychological effects, including increased levels of anxiety and depression. Most research using self-reported data highlights the pandemic’s impact on the psychological well-being of females, whereas data for mental health emergency presentations may reflect the impact on male mental health more accurately. We analyzed records of male mental health emergencies occurring in the East Midlands of the United Kingdom during the first national “lockdown.” We computed two binary logistic regression models to (a) compare male mental health emergencies occurring during “lockdown,” 2020 (5,779) with those occurring in the same period in 2019 (*N* = 4,744) and (b) compare male (*N* = 5,779) and female (*N* = 7,695) mental health emergencies occurring during “lockdown.” Comparisons considered the characteristics of mental health emergencies recorded by ambulance clinicians (Primary Impressions), and the socioeconomic characteristics of communities where emergencies use the Index of Multiple Deprivation. We found that during “lockdown,” male emergencies were more likely to involve acute anxiety (odds ratio [OR]: 1.42) and less likely to involve intentional drug overdose (OR: 0.86) or attempted suicide (OR: 0.71) compared with 2019. Compared with females, male emergencies were more likely to involve acute behavioral disturbance (OR: 1.99) and less likely to involve anxiety (OR: 0.67), attempted suicide (OR: 0.83), or intentional drug overdose (OR: 0.76). Compared with 2019, and compared with females, males experiencing mental health emergencies during “lockdown” were more likely to present in areas of high deprivation. Understanding the presentation of male mental health emergencies could inform improved patient care pathways.

## Introduction

### Background

The novel coronavirus disease 2019 (COVID-19) pandemic and associated mitigation strategies such as “lockdown” are having adverse psychological effects on individuals in societies globally (Adams-Prassl et al., 2020; Henssler et al., 2021), including increased levels of anxiety, depression (Rajkumar, 2020), and suicidal ideation (Killgore et al., 2020). In the United Kingdom (O’Connor et al., 2021; M. Pierce et al., 2020) and elsewhere (Adams-Prassl et al., 2020; Qiu et al., 2020; Wang et al., 2021), there are reports that the psychological well-being of women has been impacted by these events more severely than for men. This narrative is echoed in a recent U.K. Parliamentary Enquiry into the mental health impacts of the pandemic on adults (Parliamentary Office of Science and Technology, 2021), which suggests that women are among the most vulnerable groups in relation to poor mental health outcomes. Almost unanimously, mental health research exploring the impacts of the current pandemic on populations relies on self-reported questionnaire data and voluntary survey participation (Adams-Prassl et al., 2020; Henssler et al., 2021; O’Connor et al., 2021; M. Pierce et al., 2020; Rajkumar, 2020; Wang et al., 2021) or involves analyzing health service use data, such as records of general practitioner (GP) or mental health specialist appointment attendance (Aragona et al., 2020; Tromans et al., 2020). These methodologies capture the mental health experience of some groups in society better than others; voluntary survey participation studies can be undermined by self-selection and nonresponse bias (Cheung et al., 2017). Similarly, diagnostic or service use data disproportionately represent groups in the population who are more likely to help-seek (Sigmon et al., 2005). Men are both less likely to participate in voluntary social surveys (Fischer et al., 2001; M. Pierce et al., 2020) and are less likely to help-seek, including accessing mental health services (Seidler et al., 2016).

While men account for three quarters of all reported suicides in the United Kingdom (Mental Health Foundation, 2020), most data sets suggest that women experience higher rates of psychological distress. Thus, it is likely that data, including service use, diagnosis, and help-seeking data, are a less accurate representation of male mental health compared with female mental health (Moore et al., 2021). Moore et al. (2021) analyzed the clinical records of ambulance clinicians attending mental health emergencies in the East Midlands of the United Kingdom and found that the number of males requiring ambulances for mental health emergencies during “lockdown” has increased compared with 2019 to a greater degree than for women. In contrast to help-seeking data, ambulance use data are less biased by self-selection because both individuals and others on behalf of a patient can call “999” in the event of a mental health emergency. Furthermore, mental health emergencies attended by ambulance staff reflect the escalation of moderate or manageable experiences of distress into acute conditions and extreme behaviors, such as suicide (Kira et al., 2019). Thus, emergency medical services (EMS) data may reflect the impact of the COVID-19 pandemic on male mental health more accurately than data that rely on self-report or help-seeking behavior. This study examines the characteristics of male mental health emergencies occurring in the East Midlands of the United Kingdom during “lockdown” in detail, including how these emergencies differ compared with prelockdown and compared with mental health emergencies experienced by women.

### Importance of the Research

For policymakers and health care providers to support populations through extraordinary events, like a pandemic or a widespread financial crisis, an accurate understanding of how those events impact all groups within the society is needed. Identifying appropriate measures and data sets that reflect the experience of different groups is central to the challenge of supporting societies through the current pandemic. Large-scale self-reported surveys about the mental health impacts of pandemic mitigation measures, like social distancing and “lockdown” in the United Kingdom (M. Pierce et al., 2020) and elsewhere (Bambra et al., 2021), emphasize the psychological distress experienced by women in particular. Other indicators related to mental health outcomes highlight the vulnerability of men, such as rates of physical health conditions, unemployment, homelessness, and mental health emergencies.

In the United Kingdom (Islam et al., 2020), the United States (Griffith et al., 2020), and elsewhere (Bambra et al., 2021), men are more likely to contract and die from COVID-19. This trend reflects wider sex-related health inequalities. Although females tend to report higher rates of physical illness (Bambra et al., 2021), males experience a lower life expectancy and higher burden of disease compared with females (Hawkes & Buse, 2013). Bambra et al. (2021) suggest that this phenomenon, the “Gender health paradox,” is related to social and biological health determinants as well as economic inequalities and failings of public policy. Physical morbidity, such as severe illness from infectious disease, increases the likelihood of mental illness, including depression and anxiety (Kolappa et al., 2013). Thus, susceptibility to physical illness during the pandemic may have increased the vulnerability of men to psychological distress.

Unemployment (Frankham et al., 2020; Xiong et al., 2020) and homelessness (Singh et al., 2019; Smith, 2005) are also associated with poor mental health outcomes and, compared with help-seeking, highlight the vulnerability of males. An analysis of labor and employment in 24 countries undertaken by the United Nations Conference on Trade (UNCTAD) indicates that while women’s labor was most affected in the early months of the COVID-19 pandemic, male unemployment worsened as the crisis continued, likely as a flow-on effect of drops in national trade and associated manufacturing industries (UNCTAD, Zarrilli & Luomaranta, 2021). Similarly, the rise in homelessness in the United Kingdom between March and December 2020, and the rise in temporary housing service demand, was overwhelmingly due to the impacts of the pandemic on men (Boobis & Albanese, 2020). For example, between April and June 2020, the number of rough sleepers in London rose by 77% compared with the same period in 2019, largely accounted for by single men.

Self-reported accounts of male mental health in the United Kingdom during the COVID-19 pandemic (e.g., M. Pierce et al., 2020) are inconsistent with the vulnerability of men suggested by socioeconomic measures like health, unemployment, and homelessness data. By contrast, records of ambulance attendance for mental health emergencies during “lockdown” demonstrate that the number of men experiencing severe mental health conditions has increased (Moore et al., 2021).

Ambulance data offer unique opportunities to explore the impacts of the COVID-19 pandemic on male mental health and to inform approaches to support men struggling during periods of extended social isolation, such as pandemic “lockdown” situations, as well as to support those men experiencing livelihood hardships like housing insecurity during more ordinary, nonpandemic times. Mental health emergencies reflect the culmination of multiple life stressors into severe symptoms (Kira et al., 2019; McLoughlin et al., 2021). Thus, mental health emergencies indicate an acute event as well as the earlier presence of more moderate or manageable conditions that have escalated unchecked. These data do not represent the true prevalence of mental health conditions among males during the COVID-19 pandemic. Rather, records of mental health emergencies are one method of identifying men who might benefit from access to primary care services and support navigating rapidly changing life circumstances, such as during the current pandemic.

In this study, we examine ambulance records of mental health emergencies occurring during “lockdown” in the East Midlands of the United Kingdom in more detail, including the characteristics of male and female emergencies reflected in Primary Impression records. Primary Impression records distinguish between symptoms that can reflect affective disorders, such as depression, and symptoms that suggest acute severe disturbance of perception or behavior, such as psychosis. Clinical practitioners and community groups may benefit from understanding the characteristics of acute male mental health events for formal mental health screening as well as raising awareness about male mental health risks. Furthermore, the role of ambulance services and paramedics is evolving from conveying most patients to the hospital to include referring patients to alternative services, such as mental health services (Eaton et al., 2018; Paulin et al., 2020). Elucidating the characteristics of male mental health emergencies compared with female emergencies may inform paramedic training to improve care pathways during ambulance attendance and to support paramedics to refer men to appropriate primary care services following an emergency.

Utilizing ambulance data also allows us to explore the relationship between economic vulnerability and male mental health during the pandemic. Unemployment and housing records suggest that financial hardship, which can precipitate psychological distress, has disproportionately impacted men. Ambulance records include spatial data indicating the location where “999” calls were made. We linked each unique spatial data point for “999” call locations to a score indicating the degree of deprivation in the local community, known as the U.K. Index of Multiple Deprivation (IMD), whereby lower scores indicate greater deprivation and higher scores indicate greater affluence. Thus, we used spatial data to consider whether community deprivation and affluence vary for male mental health cases occurring during “lockdown” compared with the year prior and whether deprivation and affluence within communities vary between males and females experiencing mental health emergencies during “lockdown.”

## Method

### Research Aims

The aims of the study were the following:

1. To explore the characteristics of male mental health emergencies during the first national COVID-19 lockdown’ in the United Kingdom between March 23 and July 4, 2020, compared with the same period in 2019, including whether the nature of Primary Impressions recorded by paramedics has changed and whether the socioeconomic status of communities where mental health emergencies take place has altered.
2. To elucidate differences between the characteristics of mental health emergencies experienced by males compared with females during the first national “lockdown,” including Primary Impressions and the socioeconomic status of communities where mental health emergencies occur.

### Study Design, Setting, and Population

National “lockdown” to mitigate the transmission of COVID-19 in the United Kingdom occurred between March 23 and July 4, 2020. We performed a retrospective observational analysis using data from the East Midlands Ambulance NHS Trust (EMAS), including records of Primary Impressions, indicating the preliminary diagnosis of ambulance paramedics on scene. The East Midlands is located in the Central Eastern part of England and spans an area of 15,627km^2^. The region has an estimated population of 4.8 million and includes the urban centers of Derby, Leicester, Lincoln, Northampton, and Nottingham. The research population included *all* males and females who have been attended by ambulances for mental health emergencies following “999; calls to ambulance services during ‘lockdown,” rather than a sample of these records. For each individual mental health emergency, medical professionals have recorded clinical impressions including anxiety, depression, psychosis, acute behavioral disturbance, attempted suicide, and intentional drug overdose.

### Measures

Table 1 summarizes the measures included in the research, including gender, Index of Multiple Deprivation (IMD) scores, and Primary Impressions. Primary Impressions categories are determined based on the professional judgment of a trained paramedic clinician as well as the mental health history reported by the patient or other individuals who are known to the patient and are present during ambulance attendance, including family members, partners, and carers. Thus, Primary Impressions reflect the presenting symptoms of the patient during the emergency as well as medical history.

**Table 1.** Research Measures Including Those Obtained From EMAS Data Set and IMD Deciles.

Measure	Data	Source
Date	Categories: 2019, 2020	East Midlands Ambulance NHS Trust
Primary Impression	Categories: acute behavioral disturbance, anxiety, attempted suicide,^a^ depression, intentional drug overdose, sectioned under the Mental Health Act, other mental health problem^b^	
Sex	Categories: Male, female	
IMD	Decile values	https://hub.arcgis.com/datasets/communities:lower-super-output-area-lsoa-imd-2019-osgb1936

*Note.* Measures obtained from EMAS were the date of “999” call received, sex, and paramedic Primary Impressions of the mental health emergency. EMAS = East Midlands ambulance NHS trust; IMD = index of multiple deprivation; NHS = National Health Service.

a Records also distinguish between suicide attempt and suicide completion. Due to small numbers, cases of suicide completion may be recognizable to readers. Therefore, our statistical analysis does not include suicide completion.

b Other mental health problem refers to cases where the nature of the mental health emergency has not been specified by the attending paramedic and may include instances where the primary problem was unable to be diagnosed on site.

### Data Handling and Cleaning

Two subsets were extracted from the data set obtained by EMAS^1^ for the purpose of analysis. The first subset comprised 10,523 records of male mental health emergencies attended by ambulances between March 23 and July 4, 2020, and the same period in 2019. The second subset comprised 13,474 individual patient records of male and female mental health emergencies attended by ambulances between March 23 and July 4, 2020. All records contained sex and Primary Impressions.

Each ambulance record of mental health emergencies was linked to an IMD decile score via a spatial join that overlays the location of emergencies onto a spatial layer containing IMD decile scores and analyses the intersection of both data sets. An IMD decile score of 1 indicates most deprived communities, while a score of 10 indicates most affluent communities. Ambulance records contain postcode regions and IMD scores at Lower Super Output (LSO) scale. ArcGIS Pro 2.6.0 was used to join region postcodes to Lower Super Output Area codes (LSOA11CD) to produce a unique database assigning each EMAS record with an IMD decile value. This method is outlined in more detail by Moore et al. (2021) who used this approach to link ambulance records of suspected COVID-19 to spatial indexes including IMD.

### Data Analysis

A binary logistic regression was conducted to explore the characteristics of male mental health emergencies occurring during compared with before “lockdown,” including Primary Impressions and IMD scores for each location where a “999” call was made. To control for the effects of seasonality (Takei et al., 1992), we compared records collated between March 23 and July 4, 2020, with records for the same period in 2019. A second binary logistic regression was conducted to examine the characteristics of male compared with female mental health emergencies occurring during “lockdown,” including Primary Impressions and IMD scores for each location where a “999” call was made.

## Results

### Descriptive Statistics

The proportion of male mental health emergencies occurring in 2019 compared with 2020 by the clinical impressions of paramedics (Primary Impressions) and IMD decile are reported in Table 2.

**Table 2:** The Number and Proportion (%) of Male Mental Health Emergencies Occurring Between March 23 and July 4 in the Years 2019 and 2020, by Primary Impression and IMD Decile.

Factor	2019		2020	
	*n*	%	*n*	%
Primary Impressions				
Other Mental Health Problem	739	16	843	15
Acute Behavioral Disturbance	88	2	133	2
Anxiety	1,488	31	2,396	42
Attempted Suicide	495	10	402	7
Depression	822	17	882	15
Intentional Drug Overdose	1,003	21	982	17
Transport under the MHA	109	2	141	2
IMD Decile				
Decile 1	835	18	1,171	20
Decile 2	747	16	817	14
Decile 3	401	9	545	9
Decile 4	635	13	731	13
Decile 5	549	12	574	10
Decile 6	408	9	513	9
Decile 7	293	6	345	6
Decile 8	235	5	299	5
Decile 9	388	8	453	8
Decile 10	253	5	331	6

*Note.* Proportions (%) are rounded; therefore, the totals of Primary Impressions are below 100%. IMD = index of multiple deprivation; MHA = mental health act.

The proportion of male compared with female mental health emergencies occurring between March 23 and July 24, 2020, by the clinical impression of paramedics (Primary Impression) and IMD decile are reported in Table 3.

**Table 3.** The Number and Proportion (%) of Male and Female Mental Health Emergencies Occurring Between March 23 and July 4, 2020, by Primary Impression and IMD Decile.

Factor	Males		Females	
	*n*	%	*n*	%
Primary Impressions				
Other Mental Health Problem	843	15	883	12
Acute Behavioral Disturbance	133	2	70	<1
Anxiety	2,396	42	3,767	49
Attempted Suicide	402	7	508	7
Depression	882	15	989	13
Intentional Drug Overdose	982	17	1,343	18
Transport under the MHA	141	2	135	2
IMD Decile				
Decile 1	1,171	20	1,206	16
Decile 2	817	14	1,101	14
Decile 3	545	9	711	9
Decile 4	731	13	968	13
Decile 5	574	10	848	11
Decile 6	513	9	649	8
Decile 7	345	6	589	8
Decile 8	299	5	425	6
Decile 9	453	8	739	10
Decile 10	331	6	459	6

*Note.* Proportions (%) are rounded. As a result, the total for females exceeds 100%. IMD = index of multiple deprivation.

### Characteristics of Male Mental Health Emergencies Occurring During “Lockdown” in 2020 Compared With the Same Period in 2019

A binary logistic regression analysis was conducted to investigate the characteristics of male mental health emergencies occurring between March 23 and July 4, 2020, and the same period in 2019. In total, 4,744 male mental health emergencies occurred during this period in 2019 and 5,779 occurred during “lockdown” in 2020. The analysis examined whether the nature of Primary Impressions recorded by paramedics has changed, and the socioeconomic status of communities where mental health emergencies take place has changed. The proportion of overall cases occurring in 2019 (45%) and 2020 (55%) was approximately symmetrical. Thus, the model cutoff was set to 0.5. The results indicate that three categories of Primary Impressions (anxiety, attempted suicide, and intentional drug overdose) and five categories of IMD Decile (Deciles 2, 4, 5, 7, and 9) were significant predictors of male mental health emergencies occurring during the first national “lockdown” compared with the same period in 2019, (χ^2^ = 171.89, *df* = 15, *p* < .001). The model correctly predicted 78.5% of cases occurring during “lockdown” and 29.8% of cases occurring in 2019, giving an overall prediction accuracy of 56.6%. The Nagelkerke *R*^2^ value for the model was .02.

Table 4 displays the binary logistic regression results for all independent variables included in the model. The reference categories for categorical variables in the model were as follows: “Other Mental Health Problem” for Primary Impressions, and “Decile 1” for IMD decile. Both reference categories were the most common for each measure. Decile 1 represents regions of greatest deprivation. Compared with male mental health emergencies occurring in 2019, cases occurring during “lockdown” in 2020 were more likely to be associated with anxiety and less likely to involve attempted suicide and intentional drug overdose. A trend related to acute behavioral disturbance was also observed (*p* = .058). Furthermore, mental health emergencies occurring during “lockdown” were less likely to occur in communities characterized by IMD Deciles 2, 4, 5, and 7 compared with Decile 1, the most deprived IMD decile. Overall, the strongest predictor of male mental health emergencies occurring in “lockdown” compared with the same period in 2019 was anxiety (odds ratio [OR]: 1.42).

**Table 4:** Binary Logistic Regression for Predicting Male Mental Health Emergencies Occurring During “Lockdown” Compared With the Same Period in the Year Prior to Lockdown (2019).

Variable		*B*	*SE*	Wald	*df*	Exp(*B*)	95% CI
Primary Impressions	Acute Behavioral Disturbance	.28	.15	3.6	1	1.32	[.99, 1.76]
	Anxiety	.34	.06	33.13	1	1.42*	[1.26, 1.6]
	Attempted Suicide	−.34	.08	16.61	1	.71*	[.6, .84]
	Depression	−.06	.07	.77	1	.94	[.82, 1.08]
	Intentional Drug Overdose	−.15	.07	4.97	1	.86**	[.75, .98]
	Transport Under the MHA	.15	.14	1.25	1	1.17	[.89, 1.53]
IMD Deciles	IMD decile 2	−.25	.07	13.25	1	.78*	[.68, .89]
	IMD decile 3	−.03	.08	.11	1	.97	[.83, 1.14]
	IMD decile 4	−.2	.07	7.85	1	.82*	[.71, .94]
	IMD decile 5	−.31	.08	16.52	1	.74*	[.64, .85]
	IMD decile 6	−.13	.08	2.51	1	.88	[.75, 1.03]
	IMD decile 7	−.21	.09	5.08	1	.81**	[.68, .97]
	IMD decile 8	−.13	.1	1.73	1	.88	[.72, 1.07]
	IMD decile 9	−.21	.08	.6.18	1	.81**	[.69, .96]
	IMD decile 10	−.1	.1	1.03	1	.91	[.75, 1.1]

*Note.* Predictor variables include Primary Impressions and IMD Decile. IMD = index of multiple deprivation.

* Statistically significant at *p* < .01. **Statistically significant at *p* < .05. ***Trend at *p* < .1.

### Differences Between Male and Female Mental Health Emergencies Occurring During “Lockdown” in 2020

A binary logistic regression analysis was conducted to explore differences between male and female mental health emergencies occurring during the first national “lockdown,” including Primary Impressions and the socioeconomic status of communities where mental health emergencies occur as measured by IMD decile. The overall proportion of female cases (*N*= 7,695, 58%) and male cases (*N* = 5,779, 42%) was slightly asymmetrical. Thus, a Receiver–Operating Characteristic (ROC) Curve was computed to determine the optimal cut-off point which was found to be .4. The results of the regression model indicated that four categories of Primary Impression (Acute Behavioral Disturbance, anxiety, attempted Suicide, and intentional drug overdose), and all categories of IMD decile were significant predictors of male compared with female mental health emergencies occurring during “lockdown” (χ^2^ = 196.29, *df* = 15, *p* <.001). The model correctly predicted 65.6% of male mental health emergencies and 43.2% of female mental health emergencies occurring during “lockdown,” giving an overall prediction accuracy of 52.8%. The Nagelkerke *R*^2^ value for the model was .02.

Table 5 displays the binary logistic regression results for all independent variables included in the model. The reference categories for categorical variables included in the model were “Other Mental Health Problem” for Primary Impressions and “Decile 1” for IMD deciles, representing regions of greatest deprivation. Compared with females, male mental health emergencies were more likely to be associated with acute behavioral disturbance (ABD) and less likely to be associated with anxiety, attempted suicide, and intentional drug overdose. Male mental health emergencies were also less likely to be associated with all IMD deciles compared with Decile 1, indicating that male mental health emergencies are occurring in the most deprived communities. Overall, the strongest predictor of male compared with female mental health emergencies (OR: 1.99) was ABD.

**Table 5.** Binary Logistic Regression for Predicting Male Compared With Female Mental Health Emergencies Occurring During “Lockdown,” Including Primary Impressions and IMD Decile.

Variable		*B*	*SE*	Wald	*df*	OR	95%, CI
Primary Impressions	Acute Behavioral Disturbance	.69	.16	19.45	1	1.99*	[1.47, 2.7]
	Anxiety	−.4	.06	53.1	1	.67*	[.6, .75]
	Attempted Suicide	−.19	.08	5.2	1	.83**	[.71, .75]
	Depression	−.07	.07	1.2	1	.93	[.82, 1.1]
	Intentional Drug Overdose	−.27	.06	17.99	1	.76*	[.67, .86]
	Transport Under the MHA	.1	.13	.61	1	1.11	[.86, 1.4]
IMD Deciles	IMD decile 2	−.27	.06	18.22	1	.77*	[.68, .87]
	IMD decile 3	−.24	.07	11.09	1	.79*	[.69, .91]
	IMD decile 4	−.25	.06	14.81	1	.78*	[.69, .89]
	IMD decile 5	−.36	.07	27.81	1	.7*	[.61, .8]
	IMD decile 6	−.2	.07	7.59	1	.82*	[.71, .94]
	IMD decile 7	−.5	.08	40.06	1	.6*	[.52, .71]
	IMD decile 8	−.3	.09	12.01	1	.74*	[.63, .88]
	IMD decile 9	−.44	.07	36.2	1	.65*	[.56, .74]
	IMD decile 10	−.29	.08	11.76	1	.75*	[.64, .89]

*Note.* CI = confidence interval; IMD = index of multiple deprivation; OR = odds ratio.

* Statistically significant at *p* < .01. **Statistically significant at *p* < .05.

## Discussion

The state of emergency and extra-ordinary measures to contain contagion transmission precipitated by the COVID-19 pandemic, such as “lockdown,” has exposed weaknesses in healthcare systems globally and presented challenges for identifying vulnerable groups (Marmot et al., 2020). Most research into the impacts of “lockdown” on psychological well-being relies on self-reporting and help-seeking data (Adams-Prassl et al., 2020; Aragona et al., 2020; Henssler et al., 2021; O’Connor et al., 2021; M. Pierce et al., 2020; Rajkumar, 2020; Tromans et al., 2020; Wang et al., 2021; Wiu et al., 2020) and thus tends to underrepresent some of the most vulnerable groups in societies, including males who are less likely to participate in voluntary social surveys or access mental health services (Cheung et al., 2017; Fischer et al., 2001; Seidler et al., 2016). Ambulance records of mental health emergencies may be less affected by self-selection bias, and therefore offer a novel opportunity to explore the impact of the pandemic on male mental health (Moore et al., 2021).

We used ambulance data to explore the characteristics of male mental health emergencies during “lockdown,” including the nature of emergencies and the socioeconomic conditions of communities where emergencies occur, and to consider differences in emergencies and socio-economic characteristics between males and females. In the following, we consider how our results may elucidate the impact of the COVID-19 pandemic on male mental health and suggest some implications for supporting vulnerable males during extended periods of social isolation associated with “lockdown” as well as other more ordinary circumstances in which males may experience social isolation.

### Male Mental Health Emergencies During “Lockdown”

Our analysis indicates that males are more likely to be attended by ambulances for acute anxiety during “lockdown” compared with the same period in 2019. The impact of “lockdown” on female anxiety has been well documented (Adams-Prassl et al., 2020; O’Connor et al., 2021; M. Pierce et al., 2020; Qiu et al., 2020; Wang et al., 2021). However, prior research is likely to underrepresent the psychological experience of males due to self-selection bias. Similarly, there is some evidence that anxiety is underreported by males (Gallacher & Klieger, 1995; K. A. Pierce & Kirkpatrick, 1992), resulting in underdiagnosis (Vanderminden & Esala, 2019). Ambulance data offer an alternative perspective on male anxiety; “lockdown” has had a profound effect on populations, and males are not immune to psychological distress. Taken together, wider research about help-seeking and our findings are an indication that while males experience acute psychological distress, they may underreport their experiences. However, our data are not representative of true rates of acute male anxiety in the East Midlands population. Rather, our analysis highlights the overrepresentation of acute male anxiety cases attended by ambulances.

In addition, a nonsignificant trend (*p* < .1) was observed for ABD, suggesting that the proportion of ambulance attendance for males experiencing aggressive behavioral symptoms may have been marginally higher (<1%) during “lockdown” compared with 2019. Certainly, the frequency of ABD cases increased by nearly 34% during “lockdown” compared with 2019. The importance of ABD presentations is discussed in detail below (*Section 4.2*).

Consistent with prior research related to suicidality (Moore et al., 2021), we found that males are less likely to be attended by ambulances for attempted suicide and intentional drug overdose during “lockdown” compared with 2019. This trend reflects the observations of others in regions where full “lockdown” was imposed (Mourouvaye et al., 2021) compared with regions where partial or no “lockdown” was imposed, such as throughout the United States (Moutier, 2021). To preserve the anonymity of patients, we are unable to report the number or proportion of attempted suicides that resulted in suicide completion.^2^ However, we have observed that the numbers are comparable, with one more suicide completion occurring in 2019 compared with “lockdown.”

To date, the causal pathways for why suicide attempts (as opposed to completions) have decreased in the United Kingdom and elsewhere are poorly understood. Urgent qualitative community-scale research is needed to elucidate causes, in particular, to establish whether rates of suicidality are related to reduced means during “lockdown.” Suicidal behavior reflects the progression of an individual through a “developmental cascade”; biological factors may predispose an individual to impulse-aggressive behaviors, while specific life stressors can act to “trigger” suicidality (Turecki, 2005). Male suicide completion tends to involve violent means, such as hanging and jumping from height in the United Kingdom (Cutcliffe, 2003) and by shooting in the United States (Andrés & Hempstead, 2011). Reduced access to means during “lockdown” in the United Kingdom and elsewhere may explain the difference between suicidality in these regions compared with the United States where the sale of guns increased by 85% at the beginning of the COVID-19 pandemic (Moutier, 2021) and “lockdown” was much less stringently enforced (Ren, 2020). It is possible that male suicidal behavior may increase in the longer term as life triggers accumulate and access to means increases.

Our results also suggest that male mental health emergencies attended by ambulances during “lockdown” were more likely to occur in communities characterized by the lowest IMD decile, Decile 1, compared with five of nine other deciles (Deciles 2, 4, 5, 7, and 9). Decile 1 reflects communities of greatest deprivation. The Index of Multiple Deprivation aggregates seven domains, including education, which is commonly associated with physical and mental health literacy (Kaneko & Motohashi, 2007; Van Der Heide et al., 2013); individuals from more deprived areas tend to be less knowledgeable about recognizing symptoms and managing mental health conditions, and less likely to help-seek to address symptoms before escalation to more serious conditions (Fox et al., 2001; Uddin et al., 2019). Thus, during “lockdown” male mental health emergencies disproportionately occurred in areas that are likely to be characterized by low rates of education, mental health literacy, and help-seeking behavior.

However, cases occurring in Deciles 3, 6, 8, and 10 were not found to be predictors of mental health emergencies during “lockdown” compared with the previous year. In fact, the frequency of cases occurring in these deciles increased by ≥20% during “lockdown.” This suggests that while deprivation may explain the majority of male mental health emergencies, high rates of emergencies also occurred in communities characterized by affluence. This finding requires further consideration. It is likely that factors beyond socioeconomic situations impact the escalation of manageable psychological distress to acute conditions in males. These factors might include features of environmental landscapes, such as access to health-promoting services and spaces (Krefis et al., 2018), like general practitioners and green spaces that are known to promote wellbeing (Hartig et al., 2020). The association between high rates of male mental health emergencies during “lockdown” and landscape-scale factors, such as access to services and degree of rurality compared with urbanization, is explored in detail in a companion piece appearing in this special edition (*Characterising unusual spatial clusters of male mental health emergencies occurring during the first national COVID-19 “lockdown” in the East Midlands region, UK: a geospatial analysis of ambulance 999 data*). Overall, our results indicate that males have experienced higher rates of acute anxiety during “lockdown.” The discrepancy between this finding and prior research (Adams-Prassl et al., 2020; O’Connor et al., 2021; M. Pierce et al., 2020; Qiu et al., 2020; Wang et al., 2021) suggests that males may not be reporting their experiences. Mental health emergencies during “lockdown” have occurred disproportionately in most deprived communities, which is consistent with associations between deprivation, education related to health literacy, and poor help-seeking behavior.

### Differing Characteristics of Male Compared With Female Mental Health Emergencies

Compared with females, male mental health emergencies were more likely to be related to ABD and less likely to involve anxiety, attempted suicide, and intentional overdose. Table 2 demonstrates that nearly twice as many males were attended by ambulances for ABD compared with females. Furthermore, cases of female anxiety and intentional drug overdose occurred approximately 30% more frequently than for males, while cases of attempted suicide occurred approximately 20% more frequently for females compared with males. Male mental health emergencies were also more likely to occur in regions characterized by IMD Decile 1 and less likely to occur in regions characterized by all other deciles. The strongest predictor of male emergencies compared with female emergencies was ABD (OR: 1.99).

Due to small numbers and compliance with General Data Protection Regulation (GDPR) to preserve patient anonymity, we are unable to report the frequency or proportion of attempted suicide cases that resulted in suicide completion. However, we observe that while the proportion of attempted suicide cases is higher for females, the rate of suicide completion is higher for males by an order of 2.5. These findings are consistent with wider research about suicidality; females attempt more often but males are more likely to complete (Freeman et al., 2017; Mościcki, 1994). Furthermore, females are more likely to attempt by nonviolent means (Henderson et al., 2005), primarily by overdose, while males tend to use more violent means (Kim et al., 2003; McGirr et al., 2008).

High rates of male ABD are also consistent with prior research about impulsive-aggressive behavior. Patients presenting to emergency departments with ABD are predominately male (Oliver et al., 2019), usually require police escort as well as conveyance by ambulance, and tend to need long hospital stays with security presence to protect hospital staff from violent behavior (Lovett et al., 2022). Acute behavioral disturbance is typically characterized by dysregulation, impulsivity, and aggression (Stevenson & Tracy, 2021). A growing body of evidence supports the role of aggressive behavior in suicide risk (Dumais et al., 2005; Freed, 1975; Spirito & Esposito-Smythers, 2006), particularly for males (Turecki, 2005). It is possible that our results point to the dysregulation of male behavior during “lockdown,” a common risk factor for male compared with female suicide. Our study focuses primarily on male mental health and does not consider how female conditions may have changed because of “lockdown.” However, it is likely that the pandemic has exacerbated preexisting inequalities and behavioral differences within societies and between genders. Thus, our observations may reflect wider differences between males and females in more ordinary times rather than exclusively during “lockdown.”

Cases of males attended by ambulances for ABD can be viewed from two perspectives; first, the immediate risk of violence to both patient and health care staff and second, from the viewpoint that extreme behavioral dysregulation may be a “marker” for future suicidality. Females are more likely to internalize psychological distress and report experiencing emotive responses to distress, such as depression and anxiety, while males typically externalize distress, resulting in aggressive or impulsive behavior (Kramer et al., 2008; Leadbeater et al., 1999). Thus, psychologically distressed males are more likely to be reported for antisocial behavior and recorded in criminal justice systems (Stevens et al., 2004), while females are more likely to contact health services (Mackenzie et al., 2006). However, the experience and behavior of male and female groups are not homogeneous, and these trends do not represent the diversity of masculinities and femininities within society. Rather, our observations of ambulance data support some commonly observed differences between genders; compared with females, males are less likely to require ambulance attendance for emotive symptoms typical of internalizing responses to psychological distress and more likely to require ambulance attendance for externalizing behavioral symptoms of distress.

Trends related to internalizing and externalizing symptoms of mental health issues suggest some possibilities for supporting vulnerable males. Cutcliffe (2003) describes suicide as a trajectory along which health care providers have “windows of opportunity” for preventive intervention. A study conducted in Wales examined all cases of suicide between 2000 and 2017 and found that 75% of suicide victims had had contact with primary or secondary health care services, such as general practitioners (GPs), at least once in the weeks preceding suicide completion. However, while 78% of suicides were committed by males, a considerably higher proportion of females had contact with services compared with males. Prior contact with health care services is one “window” through which health professionals like GPs can identify individuals who may be at risk of suicidality. Given that males are less likely to actively help-seek, emergency medical data may provide a valuable opportunity for health care professionals to open doorways to intervention. However, further research is needed into the links between aggressive behavior in the context of ambulance use, and suicidality.

Our analysis *also* supports wider literature suggesting that poor psychological outcomes are associated with deprivation, including unemployment and homelessness (Frankham et al., 2020; Singh et al., 2019; Smith, 2005; Xiong et al., 2020). Compared with females, males experiencing mental health emergencies attended by ambulances during “lockdown” were more likely to live in the most deprived regions of the East Midlands, represented by IMD Decile 1. A recent longitudinal study conducted in the United Kingdom found that men’s mental health is more severely impacted by employment circumstances compared with women (Kromydas et al., 2021). Over the course of the COVID-19 pandemic, unemployment has affected males in low-income occupations, such as construction, to a greater degree than males in higher earning occupations (Allas et al., 2020), with an associated disproportionate rise in unemployment benefit claims by men in most deprived neighborhoods (Trust for London, 2021). Social surveys (O’Connor et al., 2021; M. Pierce et al., 2020) capture the mental health impacts of “lockdown” on females and give health professionals the opportunity to facilitate help-seeking. Ambulance data linked to IMD deciles may reflect the psychological impact of “lockdown” and the economic consequences of the pandemic on vulnerable males.

Overall, the analyses presented here demonstrate that ambulance data offers valuable insight into male mental health during the “lockdown” circumstances associated with the COVID-19 pandemic. A central message is that males in most deprived regions of the East Midlands are overrepresented in ambulance data; mental health emergencies are disproportionally occurring for males living in deprived communities. Compared with the year previous, males experienced higher rates of acute anxiety requiring ambulance attendance. However, compared with females, males were more likely to require ambulance attendance for ABD compared with symptoms of affective disorders such as anxiety. Thus, comparing the experience of males and females may mask the true impact of the pandemic on male anxiety. Furthermore, trends related to suicidality require urgent attention to elucidate causal pathways and determine whether rates of attempted suicide during “lockdown” point to future crisis; are societies where “lockdown” was stringently enforced facing a male mental health “sleeping dragon”?

### Limitations

Factors other than community-scale socioeconomic status are likely to influence mental health outcomes during a pandemic that involves “lockdown,” such as landscape features, including access to health services (Goddard & Smith, 2001) and green spaces for recreation (Houlden et al., 2018). Our research team considers the spatial and geographical dynamics of unusually high clusters of male mental health emergencies occurring during “lockdown” in a companion piece submitted/published in this special edition (*Characterising unusual spatial clusters of male mental health emergencies occurring during the first national COVID-19 “lockdown” in the East Midlands region, UK: a geospatial analysis of ambulance 999 data*).

Help-seeking behavior varies demographically and in relation to mental health literacy. Thus, ambulance attendance represents a proportion of mental health emergencies rather than the true frequency occurring during “lockdown.” For example, not all suicides are attended by ambulances; thus, true rates in society are probably underrepresented by ambulance data. Our observations pertain specifically to rates of suicides attended by ambulances. It is likely that unreported emergencies have occurred, particularly in more deprived communities. Furthermore, people living within proximity to a hospital or other medical facility, such as community centers, may access services directly without calling an ambulance. In addition, EMAS records include the clinical assessments of trained paramedics; however, these assessments are not diagnostic. The primary impressions involved in this research include the self-reported experience of patients as well as more objective assessments of mental state made by paramedic clinicians. However, these assessments are based on the symptoms patients are experiencing during the emergency rather than an in-depth knowledge of prior medical history. Data linkage between ambulance and hospital or primary care data would be required to determine whether ambulance patients have current diagnoses of mental health conditions.

Similarly, patients presenting to emergency departments with ABD are often repeated cases. Thus, data linkage is also necessary to establish whether any cases of ABD occurring during “lockdown” are reoccurring in patients. However, a preliminary investigation of EMAS data suggests that few cases are likely to be reoccurring patients.^3^ Furthermore, from the perspective of understanding the impact of the pandemic on male mental health, and for emergency service provision, repeat cases do not negate our central findings. Individual patients who experience reoccurring acute mental health emergencies are likely to do so prior to as well as during a pandemic. Changes in the nature or frequency of repeat cases are of equal importance to changes involving unique individuals; both circumstances reflect the impact of the pandemic on vulnerable males. In addition, unique or repeat cases require the same resources for emergency service response.

### Implications for Practice and Research

Our findings suggest opportunities for clinical practice, EMS systems, and policy intervention to support vulnerable males during extended “lockdown” conditions as well as in more ordinary circumstances where males experience social isolation, such as within migrant communities. These opportunities are not limited to the study region. The East Midlands of the United Kingdom represents a microcosm of the United Kingdom in terms of socioeconomic and rural–urban dynamics. Thus, we make four observations that apply to addressing male mental health more widely.

First, the nature of male mental health emergencies during “lockdown” varies compared with before lockdown and compared with females. These changes may present challenges for paramedic practitioners, such as the increasing frequency of ABD patients. Ambulance personnel may benefit from targeted training and protective measures during national emergencies such as pandemics or financial crises. Furthermore, understanding the epidemiology of male mental health emergencies compared with female emergencies could inform triage at the point that dispatch operatives receive “999” calls to improve patient care pathways (Moore et al., In Press). However, comparing the experience of males and females without considering the objective changes to characteristics of male mental health emergencies before compared with during “lockdown” obscures the impact of the pandemic on male anxiety. Thus, both temporal and demographic analyses are useful for unpacking the understanding of the characteristics of male mental health emergencies.

Second, urgent attention is needed to elucidate the impact of “lockdown” and the continuing pandemic on male suicide rates. It is possible that the full force of more than 18 months of disrupted employment in the most deprived regions of the United Kingdom has yet to be felt. Estimates suggest that an additional 10,000 suicides related to economic deprivation occurred in Europe and North America in the 2 years following the GFC (Deady et al., 2020). Thus, medical practitioners should be alert to signs of declining well-being in male patients in subsequent years. Health professionals without mental health training may not necessarily enquire about suicidal ideation even when patients display clear markers, such as impulsive-aggressive behavior (John et al., 2020). Therefore, health professionals, such as GPs, may require additional support to identify at-risk patients. To support the endeavors of health professionals, future research should investigate the lived experience of males in the years that follow the severe period of “lockdown” and social isolation that many experienced during the initial phases of the COVID-19 pandemic.

Third, data linkage between ambulance records and hospital and GP records is needed to explore the association between ABD and other symptoms. It is possible that ambulance data captures the “developmental cascade” toward more severe mental health presentations. Identifying pathways that precede ABD, such as anxiety and the association between ABD and suicidality, could provide the window of opportunity that health care professionals desperately need to encourage vulnerable males to engage with mental health services. Botan et al. (2021) investigated the effectiveness of issuing patients attended by ambulances for hypoglycemic emergencies with educational booklets to prevent repeated ambulance use for the same condition. They found that repeated ambulance use was significantly reduced by providing patients with information about recognizing symptoms and adherence to treatment. A similar approach could be taken to provide groups experiencing acute mental health symptoms, including males with information to improve mental health literacy and encourage help-seeking. However, qualitative research is needed to establish the most effective means of communicating with vulnerable males about their mental health experiences and to develop useful narratives to combat social stigma about help-seeking (Lefkowich et al., 2017).

Finally, a more granular spatial analysis should be conducted to identify deprived regions with high rates of mental health emergencies for the purpose of intervention. Our companion piece in this special edition presents a spatial cluster analysis of male mental health emergencies in the East Midlands of the United Kingdom as a methodology for identifying vulnerable communities and explores the implications for intervention in greater detail.

## Conclusion

The COVID-19 pandemic has drawn attention to inequalities that characterize the socio-economic landscape of the United Kingdom and elsewhere. Understanding the mental health impacts of the pandemic and mitigation measures like “lockdown” is more challenging; the scope to identify vulnerable individuals and communities is determined by data sets that often underrepresent hard-to-reach groups, such as males. Social surveys and service use data reflect the experiences of groups in society who are most likely to engage with those mediums, such as females. However, the preferences for help-seeking and interacting with the research community vary demographically, and the best means of communicating with males about mental health is poorly understood. Alternative research methods and data sets are required to capture the full range of experiences within societies. Ambulance data offer novel insights into male mental health, including the increased risk of males to acute anxiety and ABD during “lockdown,” and highlight the importance of monitoring the well-being of males in deprived communities in the years following the most severe phases of the pandemic.

Addressing the impact of contagion mitigation measures on male mental health faces many challenges, including social stigma and low levels of help-seeking behavior. The analysis presented in this study is a first step toward elucidating the experience of males during extraordinary circumstances like a pandemic when economic and social systems are disrupted. Male mental health is underresearched, in part because of difficulties with measurement. Our research presents a methodology that could be used to investigate the epidemiology of male mental health more generally and to identify opportunities for intervention. We encourage EMS to develop protocols for providing males experiencing mental health emergencies with information to facilitate and normalize help-seeking and urge health professionals who may be the first point of contact for vulnerable males to remain vigilant. It is likely that the full magnitude of impacts from successive “lockdown” phases will begin to manifest in the years that follow. Coordination and cooperation between primary care and emergency services will be essential to make the most of rare “windows of opportunity” to support males experiencing psychological distress.

## Notes

1. This research was approved by the NHS Health Research Authority, IRAS ID: 264573.

2. The number of suicide completions in 2019 and 2020 was <10. In compliance with GDPR, we have excluded these data from the formal statistical analysis presented in the research.

3. Analysis involved comparing partial postcodes and patient age for cases including all cases of ABD. A small number (<10) of duplicate cases were identified for each of male and female patients experiencing ABD. However, partial postcodes represent large regions and are not geographically specific enough to determine whether these duplicate records reflect reoccurring patients. To comply with ethical requirements and GDPR all patient data were deidentified, including aggregating spatial information to postcode region. Data linkage via NHS Digital would be required to determine the proportion of duplicate records that relate to reoccurring patients.
